# Prevalence and predictors of Cervical Intraepithelial Neoplasia among HIV infected women at Bugando Medical Centre, Mwanza-Tanzania

**DOI:** 10.1186/1750-9378-8-45

**Published:** 2013-11-15

**Authors:** Lilian Kafuruki, Peter Fabian Rambau, Anthony Massinde, Nestory Masalu

**Affiliations:** 1 Department of Obstetrics and Gynecology, Bugando Medical Centre, Box 1370, Mwanza, Tanzania; 2 Department of Pathology, Catholic University of Health Sciences and Allied Sciences (CUHAS-Bugando), Box 1464, Mwanza, Tanzania; 3 Department of Oncology, Bugando Medical Centre, Box 1370, Mwanza, Tanzania

**Keywords:** *Cervical intraepithelial Neoplasia*, *HIV*, *Mwanza*

## Abstract

**Introduction:**

Cancer of the cervix rank the second most common cause of cancer related deaths among women in Sub-Saharan Africa. It is estimated that 529, 409 new cases are diagnosed annually with a mortality rate approaching 274,883 per year. Cervical Intraepithelial Neoplasia (CIN) precedes almost all cervical cancers. The incidence rate of CIN among HIV infected women is five times higher as compared to the rate in HIV negative women. The screening for cervical dysplasia and an appropriate management in women with CIN are effective methods for preventing cervical cancer. This study was done to determine the prevalence and predictors of CIN among a HIV infected women attending Care and Treatment centre (CTC) at Bugando Medical Centre (BMC).

**Methods:**

A cross sectional survey was undertaken among HIV infected women aged 18 years and above attending at BMC CTC clinic between February and March 2013. Visual Inspection with Acetic acid (VIA) was used as the screening method for detection of CIN. Socio-demographic, reproductive and clinical information was obtained from participants and the blood was collected for CD_4_ lymphocyte count. Cervical punch biopsy for histological examination was performed for those who had VIA positive test. Data were entered and analyzed using STATA Version 12.0 soft ware.

**Results:**

A total number of 95 (26.8%) participants had positive VIA test among three hundred and fifty-five (355) HIV infected women. Histology results showed; 4(4.2%) were normal, 26 (27.4%) had an inflammatory lesion, 58(61.1%) had CIN and 7(7.3%) had invasive cervical cancer. CIN was found to be associated with a history of multiple sexual partners (P<0.001), a history of genital warts (P<0.001), and a history of STI (P = 0.010).

**Conclusion:**

The Cervical Intraepithelial Neoplasia is a problem among HIV infected women. A history of multiple sexual partners, a history of genital warts, a history STI and a low baseline CD4 T lymphocyte were significant predictors for CIN. Screening for Cervical Intraepithelial Neoplasia is recommended for all women with HIV.

## Background

Cervical cancer and HIV/AIDS are major public health problems. Worldwide, cancer of the cervix is the second most common cancer among women, with 529,409 new cases and 274,883 deaths annually. The developing countries constitute 86% of the newly diagnosed cases and 88% of the deaths [1]. The age-adjusted incidence rate of cervical cancer in Tanzania is estimated to be 50.9 cases per 100,000 world standard women population, with an age adjusted mortality rate of 37.5 per 100,000 [2]. Cervical Intraepithelial Neoplasia (CIN) precedes almost all cervical cancers. A study undertaken in Conakry Guinea, reported that the incidence rate of CIN among HIV-infected women was 4-5 times higher compared to those with no HIV infection [3]. In an attempt to reduce the cervical cancer burden, the Ocean Road Cancer Institute in Dar-Es-Salaam introduced a cervical cancer screening programme using Visual Inspection with acetic acid (VIA) in different satellite clinics throughout the country [4]. Despite this, there has been a rise in the incidence of cervical cancer in Tanzania [5].

In developing countries, the prevalence of CIN among HIV infected women is reported to be very high [6]. A study in Lusaka Zambia evaluated 150 women, using the Papanicolau smear test; more than 93% had CIN with a median CD4 count of 165cells/mm^3^[6].

The high prevalence of cervical cancer in patients with advanced HIV/AIDS infection led to its inclusion among AIDS defining conditions [5]. Currently, it is recognized that HIV infected women have a higher prevalence of Human Papilloma Virus (HPV) and the risk of infection increases with the extent of immunosuppression [7]. A higher prevalence of persistent infections with multiple high risk-HPV strains contributes to a greater risk of precancerous lesions [7]. The natural history of cervical cancer follows a prolonged period of a pre-malignant disease stage, commonly described as CIN which can take as up to 10 years before the progression to invasive cancer [8]. This shows that there is sufficient time for the women to be screened for cervical cancer and receive the appropriate treatment [7]. Studies have reported that, among HIV infected women, the risk of developing cervical cancer is 10 years earlier than in the general population, with a high rate of progression to an advanced disease with a poor prognosis [9]. Other risk factors include: age, lifetime number of sexual partners (more than four), early sexual debut, high parity, immunosuppression, smoking and oral contraceptive use for more than five years [9,10].

The Papanicolau smear test is the most widely used cervical cancer screening test, having a higher sensitivity and specificity compared to other tests [11]. However, currently, Visual Inspection with Acetic acid has been advocated as an alternative screening method to the Papanicolau smear in developing countries and this test has high sensitivity and specificity [12]. The Papanicolau smear is difficult to implement in resource limited settings due to inadequate laboratories, and lack of expert specialists. It requests a greater number of visits than the VIA and therefore increase the cost. VIA provides an effective alternative method with fewer logistical and technical constraints [12]. A study conducted in 1999 in Zimbabwe showed that VIA was a valuable tool for the detection of pre-cancerous lesions with a sensitivity of 76.7% compared to 44.3% for cytology. However, the specificity of VIA was lower (64.1%) than that of cytology (90.6%). The authors also found that the cervical abnormalities detected by VIA, 75% concurred with those detected by biopsy [13].

Although numerous studies have documented the association between HIV infection and the presence of CIN, very few have assessed the associated risk factors specific to HIV infected women. The prevalence of CIN in Tanzania is quite variable, ranging from 2.9% to 38% [14,15]. A study conducted among attendees at the Makongoro Clinic in Mwanza Region found the prevalence of Squamous Intraepithelial Lesion (SIL) to be 7%; HPV was 34%, whereby 83% of HPV was of high risk oncogenic strains [16]. The high prevalence of HPV and CIN among a low risk population is a matter of great concern, which emphasizes the need to conduct a study on the prevalence and predictors of CIN among a high risk population.

Previous studies have recommended the need for cervical cancer screening among HIV infected women. However none have suggested which screening test should be used, Papanicolau smear or VIA, for the detection of premalignant cervical lesions, especially in a low resource setting [6,14,17]. This is partly due to the lack of documentation concerning screening tests and protocols for HIV infected women. The aim of the present study was to determine the number of women with biopsy proven CIN and the predictors of CIN in HIV infected women using VIA as an alternative screening method to the Papanicolau smear in a resource limited setting.

## Methods

A cross sectional study was conducted between February and March 2013 at the HIV Care and Treatment Centre at Bugando Medical Centre, which receives an average of 80 visiting HIV infected women per day [18]. The clinic attends to patients who are on anti-retroviral drugs and to those who have not started antiretroviral therapy. The exclusion criteria for participation in the study were: allergy to acetic acid, prior total hysterectomy, wedge resection of the cervix, previous abnormal Papanicolau smear, a history of cervical cancer and pregnancy.

Convenience sampling was used to select the first participant each day from CTC register book. Thereafter every third person was included systematically in the study, until the required sample was reached. In case of refusal or not meeting the inclusion criteria, the next person was selected.

Data was collected using a structured pre-tested questionnaire. The interview was conducted in the language in which the interviewee was proficient (Swahili, English, indigenous language) with the help of an interpreter when necessary. The gathered information included socio-demographic characteristics, gynecological history, obstetric history and whether she had previously been screened for cervical cancer.

Participants with a proven HIV positive diagnosis had a CD4 lymphocyte count check using a blood sample collected through venopuncture.

A pelvic/Speculum examination was performed for the recruited participants in which inspection and visualization under an adequate light source was conducted. A solution of freshly diluted 5% acetic acid was then painted to the mucosa of the cervix using a cotton swab tipped applicator. The detection of any distinct Acetowhite areas within the transformation zone was considered to be positive after 4 minutes of waiting. Punch biopsies to determine the accuracy of the cervical evaluation were performed for all participants who were VIA positive. The samples were fixed in 10% formalin, labeled with the patients study number and sent to the laboratory for histo-pathological examination. Those with abnormal histological results were referred for follow up and management at the gynecology/oncology clinic. Cryotherapy was performed on the same setting for lesions about 5 cm width or above. For clinical suspicious cervical cancerous lesions, patients were referred to a gynaecologist for appropriate care and management.

Data were double-entered in Epidata 3.1 (Epidata Association Odense Denmark) and cleaned and analyzed using STATA 12.0 (College Station, Texas, USA). Descriptive statistics were used to demonstrate the demographic data and prevalence of cervical lesions among HIV infected women using VIA. Univariate and multivariate logistic regression were carried out to analyze factors/predictors of cervical intraepithelial neoplasia. Estimation of odds ratios and their 95% confidence interval, comparing the odds of each outcome between predictor groups was also done. An association was considered significant when the p-value was less than 0.05.

## Results

A total number of 1853 HIV infected women attended at the HIV clinic of BMC between February and March 2013. Three hundred and fifty five (355) HIV infected women were eligible for the study and were screened for the cancer of cervix. The median age of the participants was 38 years (range 18-63 years). The majority of participants were from urban area (81%) and a third was married women (33%). The majority of the respondents 225 (71.8%) had primary education.

Among the women with malignant and pre-malignant cervical lesions, 44 (75%) had a sexual debut before the age of 18 years and a median number of sexual partners reported in this study was four (range 1-8). A history of genital warts and a history of STI were reported among 53(91.4%) and 56 (97%) of the women with CIN respectively. None of the participants had ever been screened for cervical cancer previously. [See Tables 1 and 2].

**Table 1:** Socio demographic, clinical and reproductive characteristics of HIV+ women screened for CIN

**Variables**	**CIN Negative (n=297)**		**CIN Positive ( n=58) (n=58)**		**Total (n=355)**	
	**n**	**%**	**n**	**%**	**n**	**%**
**Age**						
18-25 years	20	(6.7)	1	(1.7)	21	(5.9)
26-35 years	95	(32.0)	26	(44.8)	121	(34.1)
36-49 years	148	(49.8)	24	(41.4)	172	(48.4)
50+ years	34	(11.5)	7	(12.1)	41	(11.6)
**Residence**						
Urban	244	(82.2)	44	(75.9)	288	(81.1)
Rural	53	(17.8)	14	(24.1)	67	(18.9)
**Marital status**						
Single	32	(10.8)	5	(8.60)	37	(10.4)
Married	107	(36.0)	12	(20.7)	119	(33.5)
Divorced	74	(24.9)	20	(34.5)	94	(26.5)
Separated	4	(1.4)	2	(3.50)	6	(1.7)
Widowed	80	(26.9)	19	(32.8)	99	(27.9)
**Education**						
None	50	(16.8)	5	(8.6)	55	(15.5)
Primary	208	(70.0)	47	(81.0)	255	(71.8)
Secondary	32	(10.8)	4	(6.9)	36	(10.1)
College or above	7	(2.4)	2	(3.5)	9	(2.5)
**Sexual Debut**						
Less than 18 yrs	90	(30.3)	44	(75.9)	134	(37.8)
18 years and above	207	(69.7)	14	(24.1)	221	(62.2)
**Lifetime sexual partners**						
One partner	16	(5.4)	0	(0.0)	5	(4.5)
2-5 partners	235	(79.1)	21	(36.2)	256	(72.1)
5+ partners	46	(15.5)	37	(63.8)	83	(23.4)
**Condom use**						
Never	134	(45.1)	33	(56.9)	167	(47.0)
Sometimes	133	(44.8)	21	(36.20)	154	(43.4)
Always	30	(10.1)	4	(6.9)	34	(9.6)
**History of genital warts**						
Yes	83	(27.9)	53	(91.4)	136	(38.3)
No	214	(72.1)	5	(8.6)	219	(61.7)
History of STI						
Yes	203	(68.4)	56	(96.6)	259	(73.0)
No	94	(31.6)	2	(3.4)	96	(27.0)

**Table 2:** Socio-demographic, clinical and reproductive characteristics of HIV+ women screened for CIN

**Variables**	**CIN Negative (n=297)**		**CIN Positive (n=58)**		**Total (n=355)**	
	**n**	**%**	**n**	**%**	**n**	**%**
**Exposure to cigarette smoke**						
Yes	107	(36.0)	13	(22.4)	120	(33.8)
No	190	(64.0)	45	(77.6)	235	(66.2)
**Ever used contraceptives**						
Yes	108	(36.4)	21	(36.2)	129	(36.3)
No	189	(63.6)	37	(63.8)	226	(63.7)
**Parity**						
Nullipara	19	(6.4)	5	(8.6)	24	(6.8)
1-2	120	(40.4)	15	(25.9)	135	(38.0)
3-5	116	(39.1)	33	(56.9)	149	(42.0)
5+	42	(14.1)	5	(8.6)	47	(13.2)
**Baseline CD4 Count**						
Less than 200 cells/mm^3^	116	(39.1)	34	(58.6)	150	(42.3)
200 cells/mm^3^ or more	181	(60.9)	24	(41.40	205	(57.7)
**Current CD count**						
Less than 200 cells/mm^3^	25	(8.4)	7	(12.1)	32	(9.0)
200 cells/mm^3^ or more	272	(91.6)	51	(87.9)	323	(91.0)
**HIV drugs**						
Yes	229	(77.1)	49	(84.5)	278	(78.3)
No	68	(22.9)	9	(15.5)	77	(21.7)
**Years living with HIV**						
6 months – 1 year	57	(19.2)	10	(17.2)	67	(18.9)
1 year-2 years	56	(18.9)	10	(17.2)	66	(18.6)
2 years and above	184	(61.9)	28	(65.5)	222	(62.5)

Ninety-five (95) HIV infected women tested positive after the VIA test. This group was further subjected to a histo-pathological examination and results were as follows: 4 (4.2%) were normal, 26 (27.4%) had an inflammatory lesion, 58(61.1%) had CIN, whereby 31 (53.4%) had CIN1 and 27(46.6%) had CIN2/3. Seven women (7.37%) had invasive cervical cancer.

An age at first sexual intercourse of less than 18 years was associated with CIN (OR 0.14; 95%CI 0.07-0.27, P<0.001). A number of life time sexual partners of more than 1 increased the likelihood of having CIN (OR 1; 95% CI 0.06-0.21, P<0.001) and a history of genital warts was significantly associated with CIN (OR 27.33; 95%CI 10.6-70.8 P<0.001). CIN was more common in participants with a previous history of STI (OR 13; 95%CI 3.1-54.3, P<0.001). [See Table 3 and 4]

**Table 3:** Predictors of CIN on Univariate logistic regression

**Variables**		**CIN Negative (n=297)**		**CIN Positive (n=58)**		**Crude OR**	**95% CI**	**p-value**
		**N**	**%**	**N**	**%**			
Age								
18-25 years		20	(6.7)	1	(1.7)	1		
26-35 years		95	(32.0)	26	(44.8)	5.47	[0.70-42.71]	0.105
36-49 years		148	(49.8)	24	(41.4)	3.24	[0.42-25.30]	0.262
50+ years		34	(11.5)	7	(12.1)	4.12	[0.47-35.95]	0.200
Marital status								
Single		32	(10.8)	5	(8.6)	1		
Married		107	(36.0)	12	(20.7)	0.72	[0.23-2.19]	0.560
Divorced		74	(24.9)	20	(34.5)	1.73	[0.60-5.01]	0.313
Separated		4	(1.4)	2	(3.5)	3.20	[0.46-22.30]	0.240
Widowed		80	(26.9)	19	(32.8)	1.52	[0.52-4.42]	0.442
Age at 1^st^ sexual intercourse								
	Less than 18 yrs	90	(30.3)	44	(75.9)	1		
	18 years and above	207	(69.7)	14	(24.1)	0.14	[0.07-0.27]	<0.001
No. of live time sexual partners								
	One partner	16	(5.4)	0	(0.0)			
	2-5 partners	235	(79.1)	21	(36.2)	0.11	[0.06-0.21]	<0.001
	5+ partners	46	(15.5)	37	(63.8)	1		
History of genital warts								
	No	214	(72.1)	5	(8.6)	1		
	Yes	83	(27.9)	53	(91.4)	27.3	[10.6-70.8]	<0.001

**Table 4:** Predictors of CIN on Univariate Logistic Regression

**Variables**		**CIN Negative (n=297)**		**CIN Positive (n=58)**		**Crude OR**	**95% CI**	**p-value**
		**N**	**%**	**N**	**%**			
History of STI								
	No	94	(31.6)	2	(3.4)	1		
	Yes	203	(68.4)	56	(96.6)	13.0	[3.1-54.3]	<0.001
Exposure to cigarette smoke								
	Yes	107	(36.0)	13	(22.4)	1		
	No	190	(64.0)	45	(77.6)	1.95	[1.01-3.78]	0.048
Ever used contraceptives								
	Yes	108	(36.4)	21	(36.2)	1		
	No	189	(63.6)	37	(63.8)	1.01	[0.56-1.81]	0.982
Parity								
	0	19	(6.4)	5	(8.6)	1		
	1-2	120	(40.4)	15	(25.9)	0.48	[0.15-1.46]	0.193
	3-5	116	(39.1)	33	(56.9)	1.08	[0.37-3.11]	0.885
	5+	42	(14.1)	5	(8.6)	0.45	[0.12-1.75]	0.250
Years since diagnosed HIV +								
	6 months-1 yr	57	(19.2)	10	(17.2)	1		
	1-2 years	56	(18.9)	10	(17.2)	1.02	[0.39-2.63]	0.971
	More than 2 years	184	(61.9)	38	(65.5)	1.18	[0.55-2.51]	0.673
HAART initiated								
	No	68	(22.9)	9	(15.5)	1		
	Yes	229	(77.1)	49	(84.5)	1.62	[0.76-3.46]	0.216
Baseline CD4 Count								
	Less than 200 cells/mm^3^	116	(39.1)	34	(58.6)	1		
	200 cells/mm^3^ or more	181	(60.9)	24	(41.4)	0.45	[0.25-0.80]	0.007
Current CD4 count								
	Less than 200 cells/mm^3^	25	(8.4)	7	(12.1)	1		
	200 cells/mm^3^ or more	272	(91.6)	51	(87.9)	0.67	[0.27-1.6]	0.377

Exposure to cigarette smoking was more likely to increase the risk of CIN than non-exposure (OR 1.95, 95% CI 1.01-3.78, P= 0.04). The prevalence of CIN was higher among women with a baseline CD4 T lymphocytes count of less than 200 cells/mm^3^ (OR 1; 95% CI 0, 25-0.80, P=0.007).

There was no significant association between CIN and age, marital status, contraceptive use, parity or current CD4 count. The use of HAART and the number of years since HIV diagnosis were not associated with CIN [See Table 4].

The predictors which had a significant association with CIN in Univariate analysis were subjected to multivariate analysis. The predictors for CIN among HIV infected women were the number of life time sexual partners (Adjusted OR 4.06; 95% CI 1.86-8.88, P<0.001), a history of genital warts (Adjusted OR 16.6; 95% CI5.91- 47.0, P<0.001), a history of STI (Adjusted OR 7.39; 95% CI 1.67-33.66, P=0.010) and a baseline CD4 lymphocytes count of less than 200cells/mm^3^ (Adjusted OR 2.71; 95% CI 01.24-5.90, P=0.012) [ See Table 5].

**Table 5:** Predictors of CIN on Multivariate logistic regression

**Variables**		**CIN+ (n=58)**		**Adjusted OR**	**95% CI**	**p-value**
		**N**	**%**			
No. of live sexual partners						
	2-5 partners	21	(36.2)	1		
	5+ partners	37	(63.8)	4.06	[1.86-8.88]	<0.001
History of genital warts						
	No	5	(8.6)	1		
	Yes	53	(91.4)	16.67	[5.91-47.0]	<0.001
History of STI						
	No	2	(3.4)	1		
	Yes	56	(96.6)	7.39	[1.67-33.66]	0.010
Baseline CD4 count						
	200cells/mm^3^ or more	24	(41.4)	1		
	Less than 200 cells/mm^3^	34	(58.6)	2.71	[1.24-5.90]	0.012

## Discussion

In this study 95 out of 355 (26.8%) HIV infected women had cervical lesions (VIA positive) that warranted histological evaluation. This finding was lower than that observed in a study conducted by Balandya *et al* in Dar es Salaam whereby 42.2% were Aceto white positive [19]. This difference can be explained by the a relatively lower median CD4 count of their population which was 351cells/mm^3^compared to this study where the median CD4 count was 450cells/mm^3^. The lower the CD4 count and advanced stage of HIV could be responsible for a higher prevalence of cervical lesions seen in their study [19]. In the present study, the prevalence of CIN proven by biopsy was 16.0%. Our findings are consistent with a work reported by Miotti *et al* among 268 postpartum women of Malawi, whereby the prevalence of CIN was 15% [20]. Although the prevalence of CIN in this population appears to be low, it is within the range described in similar publications in Africa [6,21]. A Botswana study, among HIV infected women using VIA showed a prevalence of 11%, and another study among HIV infected women in Senegal showed a prevalence of 10% [22,23]. In Africa, a high prevalence of CIN (76%) among HIV infected women has been observed in Lusaka Zambia [6]. The probable explanation could be a HIV induced immune-suppression as the median CD4 count was low (165 cells/mm^3^) compared to our study in which the median baseline CD4 count and current CD4 count were 269 cells/mm^3^ and 450 cells/mm^3^ respectively. Other studies in Tanzania reported a higher CIN prevalence of 32% and 38% [15,17]. In those studies, conventional cytology was used as a screening method, and biopsy was not done.

The prevalence of CIN in the present study was higher than that found in a study by Mayaud *et al* among antenatal clinic attendees at the Makongoro clinic Mwanza which was 7%. The difference can be explained by the fact that Mayaud’s study involved the general population at the clinic while the present study involved newly diagnosed HIV participants and those who were in follow up for HIV status in tertiary hospital [16]. VIA was offered to 355 women and 26.8% of these women had VIA positive results. This was similar to a study by De Vuyst *et al* among 653 women attending Nairobi family planning clinic, whereby 27% women had VIA positive results [24]. The histological results showed 53.4% had CIN1 and 46.6% had CIN2/3, and cervical cancer in 7.4% women, while in the study done by De Vuyst *et al* CIN1 was seen in 25%women, CIN 2/3 in 38% women and invasive cancer in 4% women [24]. In our study the frequency of chronic cervicitis with feature of HPV among HIV infected women was 27.4%, this is similar to a study by Mwakigonja *et al* which showed a cervicitis in 28%, which was dominated by feature of Chlamydia trachomatis [15].

The histological results of CIN and invasive cancer seen in this setting emphasize the importance of introducing a screening programme for cervical cancer in HIV infected women. Despite of being at risk of CIN, none of the participants in this study had previously been screened for cervical cancer. This is a major concern in many developing countries including Tanzania where cervical cancer screening is largely unavailable, leading to late presentation with advance cervical cancer [14].

The majority of women acquired HIV infections within a few years of sexual debut. In this study, an early sexual debut appeared to be associated with the development of CIN but was not statistically significant. This concurs with other studies done in Tanzania whereby an early sexual debut was not directly associated with CIN [14,17]. In a study by Matasha *et al* involving female pupils in Mwanza, 68% of girls had an early sexual debut [25]. An earlier age of sexual debut implies a longer period of sexual activity and a higher likelihood of having many sexual partners [26]. Having multiple sexual partners exposes a woman to factors predisposing to CIN such as HIV and STI. This was supported by a study done among Nairobi prostitutes that showed that women with multiple sexual partners had an increased risk of developing CIN [27]. A similar trend was seen in the current study, whereby women with many sexual partners (more than two) had more CIN compared to women with few number of sexual partners with significant difference (P< 0.001).

Co-infection with a STI like *Chlamydia trachomatis*, herpes simplex or genital warts in the presence of HPV increased the risk of CIN by causing inflammation which facilitates HPV persistence and hence cervical lesion and carcinogenesis [28,29]. In the present study, a previous history of STI, including genital warts, was associated with CIN. A previous history of STI was reported to be a cofactor needed for the progression of HPV infection to cervical dysplasia and subsequently into cancer. Studies suggest that HIV induced immunosuppression leads to inability to control the HPV expression, hence the persistence of HPV infection and the development of cervical lesions [30]. This study showed that genital warts were associated with an increase risk of CIN. A higher prevalence of HPV infection in Mwanza, found in the study by Mayaud *et al* could be a major contributor to the increased prevalence of genital warts which was found among HIV infected women in this study. Genital warts are caused by persistent low risk types of HPV infection; a history of genital warts in the absence of detectable virus still increases the risk of CIN, as shown by other studies [16,31].

In the current study, women whose baseline CD4 count was less than 200 cells/mm^3^ were more likely to have CIN than those patients with a CD4 count of more than 200cells/mm^3^. Previous studies have consistently demonstrated that the amount of CD4 count of less than 200 cells/mm^3^ is a predictor for having or developing CIN [14,17]. A study done by Wright *et al* in New York in 1994 showed that an age of more than 35 years was associated with a risk of CIN. In the current study, the association between age and CIN was not statistically significant. The same conclusion was reached seen in other studies conducted in Kilimanjaro and Dar es Salaam [14,17].

Although we did not find a significant association between marital status and risk of developing CIN in the present study, a study done in Kilimanjaro showed that single women had a tendency to have multiple sexual partners, a factor which contributed to CIN [17].

Smoking and contraceptive use have been reported to be established cofactors for HPV progression to cervical lesion. Carcinogens produced by tobacco are thought to decrease the immunity in the cervix, thus facilitating HPV persistence [30]. Furthermore, contraceptive hormones increase the expression of HPV genes in the cervix and facilitate HPV persistence [30]. In the present study, the association between CIN and those who have been ever exposed to cigarette and ever used contraception was not significant. Similarly, there was no difference in CIN prevalence between Non ART and ART user. This finding is contrary to a study done by Mwakigonja *et al* which noted that the frequency of CIN and invasive cancer were higher in women who were non HAART than those who had been on HAART. Initiation of HAART has shown to have an impact on reducing the incidence and progression of CIN [15]. A study by Balandya *et al* showed that women who had been on ART for more than 1 year had more abnormal cervical changes on VIA compared to those who were on ART for less than a year. The probable explanation is that despite an increased CD4 count with reconstitution of immunity there is no clearance of HPV in the cervix [19]. In the current study, the duration since HIV diagnosis, was not associated with CIN, which is contrary to a study done by Kreiss *et al* which showed that women living with a HIV infection for more than two years were more likely to carry high risk HPV infections, which could result in CIN, compared to women who had been HIV infected for less than a year [27].

## Conclusion

CIN remains a problem among HIV infected women. A history of multiple sexual partners, a history of genital warts, a history STI and a baseline CD4 T lymphocyte of less than 200cells/mm^3^ are significant predictors for CIN. Screening for CIN is recommended for all women with HIV.

### Ethical consideration

Ethical clearance was sought from the Department of Obstetrics and Gynecology and the Joint CUHAS/BMC Research, Ethics, and Publication Committee.

### Consent

An Informed consent was requested from the participants after explaining the aims of the study. For literate women, the consent information was provided followed by a consent form which each participant was required to sign to signify her consent. For illiterate women, the consent information sheet was read in full and participants were requested to place a thumb print on the consent form to signify their acceptance to participate in the study. It was explained that participation was voluntary and those declining to participate were still entitled to the standard care provided to all women at the CTC.

## Competing interests

The authors declare that they have no competing interest. All the authors read through the article and agreed.

## Authors’ contributions

LK provided major contributions in concept, study design, literature review, data collection, data entry, data analysis and writing of manuscript. PR contributed to the concept, development of proposal, review of histological slides and writing of manuscript. AM contributed to the development of the proposal and writing of manuscript. NM contributed to the development of the proposal and writing of manuscript. All authors read and approved the final manuscript.

## Author information

First Author, Lilian Kafuruki.

Co Authors, Peter Fabian Rambau (Corresponding author), Anthony Massinde, Nestory Masalu.
